# QbD-guided phospholipid-tagged nanonized boswellic acid naturosomal delivery for effective rheumatoid arthritis treatment

**DOI:** 10.1016/j.ijpx.2024.100257

**Published:** 2024-05-19

**Authors:** Poonam Usapkar, Suprit Saoji, Pradnya Jagtap, Muniappan Ayyanar, Mohan Kalaskar, Nilambari Gurav, Sameer Nadaf, Satyendra Prasad, Damiki Laloo, Mohd Shahnawaz Khan, Rupesh Chikhale, Shailendra Gurav

**Affiliations:** a Department of Pharmacognosy, Goa College of Pharmacy, Panaji, Goa University, Goa-403 001, India; b Department of Pharmaceutical Sciences, R. T. M. Nagpur University, Nagpur, Maharashtra- 440 033, India; c Department of Pharmacology, PDEA's S. G. R. S. College of Pharmacy, Saswad, Maharashtra-412 301, India; d Department of Botany, A.V.V.M. Sri Pushpam College (Autonomous), (Affiliated to Bharathidasan University), Poondi 613 503, India; e Department of Pharmacognosy, R.C. Patel Institute of Pharmaceutical Education and Research, Shirpur, Maharashtra- 425 405, India; f Department of Pharmacognosy, PES's Rajaram and Tarabai Bandekar College of Pharmacy, Ponda, Goa University, Goa-403401, India; g Bharati Vidyapeeth College of Pharmacy, Palus 416310, Maharashtra, India; h Department of Pharmacognosy, Girijananda Institute of Pharmaceutical Science, Girijananda Chowdhury University, Azara, Guwahati 781017, India; i Department of Biochemistry, College of Science, King Saud University, Riyadh, Saudi Arabia; j UCL School of Pharmacy, 29−39 Brunswick Square, London WC1N 1AX, United Kingdom

**Keywords:** *Boswellia serrata*, Rheumatoid arthritis, Phytosome, Phospholipid-complex, TNF- α

## Abstract

Studies have reported the potential role of Boswellic acids (BAs), bioactive pentacyclic triterpenes from *Boswellia serrata* (BS), in treating rheumatoid arthritis (RA). However, poor water solubility and limited oral absorption are restricting factors for its better therapeutic efficacy. Based on these assumptions, the current study aimed to develop naturosomal delivery of BAs to boost their extremely low bioavailability, colloidal stability, and water solubility. Nanonized naturosomes were developed and subsequently analyzed to show their physicochemical and functional features employing the quality-by-design approach. The solubility analysis of Boswellic acid naturosomes revealed a 16 times improvement in aqueous solubility compared to BS extract (BSE). The zeta potential and dynamic light scattering findings of BSE naturosomes (BSENs) have demonstrated their colloidal stability with regulated nano-size particles. Additionally, compared to BSE (⁓31%), *in-vitro* dissolution experiments showed that >99% of pentacyclic triterpenes were released from BSENs. Studies on *ex-vivo* permeation showed that BSENs' permeation (>79%) significantly improved over BSE's (⁓20%). *In-vivo* efficacy studies using CFA-prompted arthritis in rodents showed a critical expansion in body wt and an undeniable reduction in paw thickness, paw volume, and TNF-α treated with BSEN compared to the arthritis control and BSE-treated group. These findings suggest that BSENs can help treat RA drugs by demonstrating their efficacy in further clinical research to validate the significant improvements.

## Highlights

• Boswellic acid naturosomes (BAN) were prepared using the solvent evaporation method.
• The physicochemical characterization revealed the effective development of the BAN.
• TNF-α levels in FCA arthritic animal models were reduced in BAN-treated animals.
• BAN exhibited a better anti-arthritic effect with enhanced bioavailability in rats.

## Graphical abstract

**Figure:**
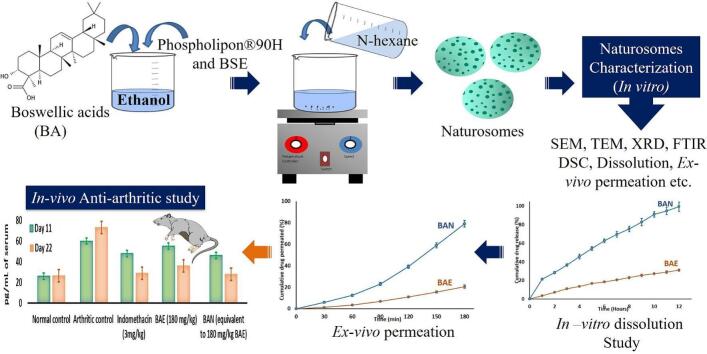

## 1 Introduction

A chronic, inflammatory, and autoimmune condition known as rheumatoid arthritis (RA) is thought to affect 0.24 to 1% of people worldwide. It is characterized by discomfort, articular cartilage degeneration, and edema and inflammation around the joints (Majeed et al., 2021). Nonsteroidal anti-inflammatory drugs (NSAIDs), corticosteroids, anti-rheumatic drugs, and biological response modifiers are used in the clinical management of RA. Still, their toxicities, iatrogenic reactions, and side effects compromise the therapeutic process. As a result, there has been a lot of interest in herbs that have anti-RA activity as possible safe alternatives to or supplements to anti-inflammatory drugs (Khayyal et al., 2018).

A branching tree known as *Boswellia serrata* Roxb. (BS), a member of Burseraceae, thrives in the arid parts of India and the Middle East. The plant has a variety of uses, including those in food, medicine, materials, and cosmetics. An olibanum or frankincense-like gum resin is found in these trees. These gum resins have a long history of treating many bacterial and inflammatory illnesses (Khayyal et al., 2018; Suther et al., 2022). Di Lorenzo et al. (2013) have reported BS as one of the plant food supplements used to treat inflammatory conditions. Based on their pilot study, Italiano et al. (Italiano et al., 2020) have proposed BS-containing food supplements to improve the quality of life of osteoarthritic patients. The extract of BS gum resin (BSE) has shown anti-RA properties. Additionally, BSE has also been demonstrated to have anti-inflammatory cytokine inhibition potential. In rat models of collagen-induced arthritis, it decreased interleukin-1 (IL − 1), tumour necrosis factor (TNF), and interferon (IFN) and boosted interleukin-10 (IL − 10) production. These cytokines are critical in persistent inflammation and tissue destruction during the progression of RA (Kumar et al., 2019).

The oleo gum resins of *Boswellia* species include triterpenes known as boswellic acids (BAs). There are about twelve distinct pentacyclic triterpenes (BAs) known. 3-acetyl−11-keto-beta-boswellic acid (AKBBA) and beta-boswellic acid (BBA) have attracted much pharmacological attention. AKBBA inhibits the leukotriene-mediated inflammatory pathways and 5-lipoxygenases (5-LO). AKBBA also lowers the activity of cyclooxygenase−1 in human platelets, NF-κB induction, inflammation-promoting cytokines, and leukotriene inhibition (Roy et al., 2019). A preliminary pharmacokinetic test revealed a poor bioavailability observed with one of the pentacyclic triterpenes of BAs, particularly 3-acetyl−11-keto-boswellic acid (AKBA). The systemic bioavailability of BAs is restricted since they are steroidal (lipophilic) and cannot dissolve into an intestinal fluid (Sharma et al., 2010). Commercially accessible boswellic acid extracts can be found in pharmaceutical and nutraceutical products. However, there is currently no research on the extract's low water solubility, nor are there any methods to increase the solubility and bioavailability regarding the treatment of RA.

Several strategies have been proposed to address phytoactives' poor solubility and bioavailability, including innovative formulations such as emulsions, liposomes, nanoparticles, chemical structural modification, and prodrug delivery. A possible method for enhancing phytoactives' bioavailability is a naturosomal drug delivery, i.e., a phospholipid carrier technique. Compared to traditional herbal medications or extracts, naturosomes are more readily absorbed due to the nanonized complex tagged with phospholipid, thus increasing the bioavailability, reducing the dose, and sustaining the duration of action. Recent studies have also demonstrated the efficacy of complexing phytoactives with dietary phospholipids to boost their bioavailability and, thus, therapeutic efficacy (Gurav et al., 2022; Metkari et al., 2023; Saoji et al., 2017; Saoji et al., 2022; Saoji et al., 2016b). BSE is, therefore, a suitable option for the development of naturosomes.

Given this, the current study aimed to determine if the anti-inflammatory effect of nanonized BSE would be enhanced by naturosomal drug delivery in an animal model of RA produced by Freund's Complete Adjuvant (FCA). A Quality by design (QbD) strategy optimized the formulated BSE naturosomes (BSENs) and subjected them to their physicochemical, functional, and pharmacological attributes.

## 2 Materials and methods

### 2.1 Materials

Natural Remedies Ltd., Bangalore, India, provided the standardized BSE, which included ∼30% AKBA. Analysis using high-performance liquid chromatography (HPLC) verified the BSE's identification. The German company Lipoid, Ludwigshafen, graciously donated hydrogenated soy phosphatidylcholine (Phospholipon® 90H).

### 2.2 Analysis of the 3-acetyl−11-keto-boswellic acid (AKBA) present in BSE

The concentrations of AKBA in BSE were determined using a modified reverse-phase (RP) HPLC method (Mannino et al., 2016). The HPLC system (Shimadzu, Japan) with LC solution software was employed, equipped with a manual rheodyne sample injector, an SPD-M20A detector, and an LC-20 CE HPLC pump with gradient elution. The mobile phase was made up of (a) Water: Methanol (50:50) containing 5 mM ammonium acetate and (b) Methanol: 1-Propanol (80:20) containing 5 mM ammonium acetate (25:75, *v*/v), at a flow rate of 200 μL/min. With a detector wavelength of 250 nm at room temperature, a Micra-NPS RP18 column (33 × 8 mm, 1.5 μ porous silica) was utilized as the stationary phase. The AKBA calibration curve was generated by analyzing the concentration of the AKBA standard solution and then graphing the peak regions against concentration.

### 2.3 Preparation of BSEN

The BSEN was developed by slightly tweaking the solvent evaporation process described in our previous studies (Gurav et al., 2022; Saoji et al., 2017; Saoji et al., 2016b) and employing the QbD methodology. In a circular bottom flask measuring 100 mL, different ratios of Phospholipon®90H and BSE were used, such as 0.5:1, 2:1, or 3.5:1, and the mixture was supplemented with 40 mL of ethanol. A water bath was used to control and sustain the reaction at various temperatures, such as 30, 40, or 50 °C. Different periods, i.e., 1, 2, or 3 h, were used to conduct the reaction. A surplus of n-hexane was added while stirring to ensure a clear solution, which had been dried up to 2–3 mL. The dispersion was filtered and vacuum-dried after it had formed to eliminate any leftover solvents. The resultant BSENs were kept at room temperature for additional analysis in amber-coloured glass vials that had been nitrogen flushed.

### 2.4 QbD approach

Currently, the QbD technique is employed to create high-quality products. QbD refers to a factual, prospect-based, comprehensive, and proactive strategy for developing pharmaceuticals that begins with fixed objectives and focus on understanding the products and processes, using sound science and excellent risk management, with better control process (Dias et al., 2022; Halarnekar et al., 2023; Rarokar et al., 2021; Rodrigues et al., 2020; Rodrigues et al., 2022). With the help of a QbD-based strategy and 17 experimental trials (Design-Expert software Version 13.0- Stat-Ease Inc., Minneapolis, USA), we investigated how changing the phospholipid-to-drug ratio (X_1_, w:w), the reaction temperature (X_2_, °C), and the reaction duration (X_3_, h) affected the product's Critical Quality Attribute (CQA), and entrapment efficiency (EE) (Gurav et al., 2022). A statistical model (Eq. (1)) with interaction and polynomial components was applied to investigate the effect of independent variables on response.

(1) $$Y=b_{0}+b_{1}X_{1}+b_{2}X_{2}+b_{3}X_{3}+b_{11}{X_{1}}^{2}+b_{22}{X_{2}}^{2}+b_{33}{X_{3}}^{2}+b_{12}X_{1}X_{2}+b_{23}X_{2}X_{3}+b_{13}X_{1}X_{3}$$

Where *B*_*i*_ is the estimated coefficient for the factor Xi, *b*_*0*_ is the arithmetic mean response of the 17 runs, and Y is the dependent variable. The principal effects (X_1_, X_2,_ and X_3_) indicated the typical outcome of the incremental rise in each factor. The three variables were adjusted concurrently, and the interaction terms (X_1_X_2_, X_2_X_3_, and X_1_X_3_) demonstrated how the response changed. The polynomial terms (X_12_, X_22_, and X_32_) were added to analyze on-linearity. Tables S1 and S2 (Supplementary File) provided information about the central composite design (CCD) batches.

### 2.5 EE of BSEN

The EE, measured as the amount of AKBA trapped by the BSEN, was determined using a reported method (Tan et al., 2012). Briefly, 10 mL of chloroform was mixed with precisely weighed (100 mg) BSEN powder and analyzed using HPLC, as mentioned in Section 2.2. Eq. (2) was used to determine the prepared BSEN's EE.

(2) $$\mathrm{EE}\;\left(\%\right)=C_{t}-C_{f}/C_{t}\times 100$$

Where C_t_ = A complete concentration of BSE, C_f_ = BSE found in the filtrate.

### 2.6 Determination of AKBA content in BSEN

AKBA content in prepared BSEN was determined using the reported HPLC method as specified above and computed using the following Eq. (3);

(3) Drug content=amount of drug in the BSEN/Amount of BSEN×100%

### 2.7 Physicochemical characterization of BSEN

#### 2.7.1 Photomicroscopy

In order to conduct a microscopic examination, a suspension made up of approximately 100 mg of the BSEN was transferred to a glass tube and diluted with 10 mL of phosphate buffer saline (pH 7.4). When the suspended vesicles were put on a transparent glass slide, a microscope (Model: DM 2500, Leica Microsystems, Germany) took photomicrographs at 20× magnification (Saoji et al., 2022).

#### 2.7.2 Scanning electron microscopy (SEM)

Briefly, the BSEN was layered on double-sided carbon tape and a brass stub. With the help of the fine auto coater, palladium was coated onto the surface powder. Using SEM (Model: JFC1600, Jeol Ltd., Tokyo, Japan) with a digital camera and an increasing voltage of 10 KV, palladium-coated samples were examined (Rarokar et al., 2021; Andrade et al., 2024).

#### 2.7.3 Transmission electron microscopy (TEM)

A small amount of the BSEN was placed on a grid made of copper and negatively smeared with 2% uranic acid for the TEM examination (Make: Jeol, Model: JEM 2100) (Gurav et al., 2022).

#### 2.7.4 Fourier transform infrared spectroscopy (FTIR)

An FTIR spectrophotometer with an attenuated total reflectance (ATR) accessory (Model: IR Prestige-21, Shimadzu, Japan) was used to obtain the infrared spectra of BSE, Phospholipon®90H, and BSEN. The materials were vacuum-dried to presumably eliminate the influence of any remaining moisture before any spectra were taken. For each sample analysis, 45 scans were performed at a resolution of 4 cm^−1^ from 4500 to 400 cm^−1^ (Rarokar et al., 2021).

#### 2.7.5 Differential scanning calorimetry (DSC)

Using a differential scanning calorimeter, the tested substances (BSE, Phospholipon®90H, and BSEN) were thermally analyzed (Model: Q20, TA Instruments, Inc., New Castle, DE, USA). Dry nitrogen gas purged the area during the analysis (50 mL/min). The instrument's heat capacity and flow were calibrated using high-purity indium. Using a crimper, the samples (2.5–5 mg) were packed in aluminum pans with their covers. Each sample went through a single cycle of heating from 0 to 400 °C at a rate of 10 °C/min. The Universal Analysis program version 4.5 A, build 4.5.0.5, was used to analyze the peak transition onset temperatures of the samples (TA Instruments, Inc., New Castle, DE, USA) (Rodrigues et al., 2022).

#### 2.7.6 Powder X-ray diffraction (PXRD)

PXRD (Model: D2 Phaser, Bruker AXS, Inc., Madison, WI, USA) with a Bragg-Brentano geometry (θ/2θ) optical setup was used to assess the polymorphic state of the materials (SBE and BN). As the diffraction angle increased from 2° to 90°, 2θ angle, the samples were scanned with a step-angle of 0.2° 2θ and a count time of 0.5 s (Saoji et al., 2017).

#### 2.7.7 Particle size and zeta potential analysis

The BSEN was subjected to a photon correlation spectroscopy (PCS) study of particle size utilizing dynamic light scattering on a Zetasizer® nano (Model: Zen 3600, Malvern Instruments, Malvern, UK) fitted with a 5 mW Helium-Neon laser with an output wavelength of 633 nm. Measurements were conducted at 25 °C, a 90° angle, and a runtime of at least 40 to 80s. Based on the electrophoretic mobility of naturosomes, the zeta potential was calculated using Smoluchowski's equation (Gracias et al., 2023).

### 2.8 Functional evaluation of BSEN

#### 2.8.1 Apparent solubility analysis

Apparent solubility tests were conducted using the stated method (Gurav et al., 2022). In brief, 10 mL of water or n-Octanol was added to an excess of BSE, a physical mixture of BSE and Phospholipon®90H (PM) and BSEN in a sealed glass vial and kept at room temperature (25 ± 0.5 °C). The mixture was then stirred for 24 h, followed by centrifugation at 4000 rpm for 30 min. The supernatant was filtered using a 0.45 μm membrane filter, followed by proper dilutions with the mobile phase and subsequent analysis using the previously mentioned HPLC technique.

#### 2.8.2 Drug dissolution study

An *in-vitro* dissolution study of test drugs, i.e., BSE, PM, and BSEN, was conducted using USP type-II dissolution apparatus (Electrolab, India, TDT-06 T). Initially, a weighed amount of BSEN equivalent to 50 mg of BSE was added to the agitated dissolution medium (900 mL phosphate buffer, pH 6.8) and stirred at 100 rpm, maintaining the temperature at 37 ± 0.5 °C. 10 mL of the sample was periodically removed and replaced with an equivalent amount of fresh dissolution medium to maintain the sink condition. Finally, the withdrawn samples were filtered using a 0.45 μm membrane filter and analyzed using the reported HPLC method. DDSolver®, an add-on program, was utilized to compare the dissolution profiles (Rarokar et al., 2023).

#### 2.8.3 Ex-vivo permeability

An adult male Wistar rat (220–250 g) was given a lethal dose of thiopental (35 mg/kg, i.v.) before being cervically dislocated to harvest its intestine for experimental purposes. Per the reported procedure, *ex-vivo* permeability studies were conducted using a Franz diffusion cell and everted intestine (Dixit et al., 2012). The everted intestine was loaded with BSEN at a predetermined concentration and placed in a small pool of receptor media (50 mL). The cellular material was agitated at 37 ± 0.5 °C using a magnetic stirrer. An aliquot of 5 mL was taken at predetermined intervals for up to 8 h to determine the drug concentration, and each withdrawal was replaced with an equivalent amount of the same diffusion medium.

### 2.9 Evaluation of *in-vivo* anti-arthritic activity

#### 2.9.1 Animals

The selection of male Wistar rats weighing 220–250 g and all animal experiments were carried out with the prior approval (SGRS/IAEC/08/2019–20) of the Institutional Animal Ethics Committee registered under the Committee for the Purpose of Control and Supervision of Experiments on Animals, Government of India (Registration No. 311/PO/ReBi/S/2000/CPCSEA). All experiments were performed per the U. K. Animals Act (1986) and its accompanying recommendations, as detailed in the ARRIVE guidelines.

#### 2.9.2 Induction of adjuvant arthritis

The evaluation of their anti-arthritic potential was conducted as per the reported method by Gautam et al. (2019). Rats were induced with adjuvant arthritis using the documented procedure (Pearson, 1956). The animals were divided into five groups of six rats each (*n* = 6) and injected with 0.1 mL of FCA into the sub-plantar region of the left hind paw on day 0:

Group I - Normal control,

Group II - Arthritic control (FCA-induced arthritis),

Group III - FCA-induced arthritis + Indomethacin as a reference drug (3 mg/kg),

Group IV - FCA-induced arthritis + BSE (180 mg/kg),

Group V - FCA-induced arthritis + BSEN (equivalent to 180 mg/kg BSE).

Each test dosage was given orally 1 h before adjuvant injection and once daily for 22 days (from day 0 to day 21). By assessing the biophysical parameters, such as the paw thickness and paw volume at days 0, 11, and 22, the anti-arthritic potential of the formulated BSEN against pure BSE was evaluated.

#### 2.9.3 Measurement of body weight

On days 0, 11, and 22 of the experiment, the body weights of the animals were noted. To measure the changes in body weight across all the tested groups, the difference in body weights on days 11 and 22 was calculated (Gautam et al., 2019).

#### 2.9.4 Measurement of paw thickness

On days 0, 11, and 22, the paw's thickness was measured to evaluate the inflammation as an acute lesion on an injected limb. According to the specified formula (4), the following percentage inhibition of paw thickness was determined:

(4) $$\text{Percentage inhibition}=\left(\mathrm{Tc}-\mathrm{Tt}\right)\times 100/\mathrm{Tc}$$

Where Tc- Mean change in paw thickness of the arthritis control group, Tt- Mean change in paw thickness of the treated group.

#### 2.9.5 Measurement of paw volume

The injected limb's paw volume of the animal was measured on days 0, 11, and 22 by positioning it vertically in the plethysmometer up to the level of the lateral malleolus (Gautam et al., 2019). The difference in the initial and final paw volumes measured the paw volume reduction.

#### 2.9.6 Measurement of TNF-α

All tested groups' blood samples were collected on days 11 and 22 via the retro-orbital route. Blood was centrifuged at 1000 rpm for 10 min to separate the serum, which was further evaluated for the TNF-α concentration using an ELISA (enzyme-linked immunosorbent assay) kit (Akhtar et al., 2021).

#### 2.9.7 Histopathological study

Following blood collection, the rats were sacrificed by cervical dislocation while being anesthetized with diethyl ether. Arthritic and inflamed joints were removed from the hind paw and preserved in 10% formalin for histological examination. The joints were decalcified with 10% formic acid for 30 days (Shabbir et al., 2014, Shabbir et al., 2016). The tissues were finally encased in paraffin. Haematoxylin and eosin staining was performed on the joint segment (5 μm). The histopathological alterations in the joints, including inflammatory cells, bone erosion, and cartilage destruction, were recorded.

#### 2.9.8 Statistical analysis

One-way analysis of variance (ANOVA) was used to analyze the statistical significance of the result obtained from the FCA-induced arthritis model. Then, the Bonferroni multiple comparisons test was performed. The outcomes were reported as mean ± SEM and were deemed significant at *p* < 0.05.

## 3 Results and discussion

### 3.1 Preparation of BSEN

Our previous studies successfully developed naturosomal delivery of hesperetin, *Withania somnifera*, *Centella asiatica*, and *Bacopa monnieri* with their enhanced aqueous solubility and therapeutic efficacy (Gurav et al., 2022; Saoji et al., 2017; Saoji et al., 2022; Saoji et al., 2016b).

According to the preliminary examination of the influence of numerous factors, the phospholipid-to-drug ratio, reaction temperature, and reaction duration significantly impacted the generated naturosomes' capacity to entrap molecules. Table 1 displays the results of the EE (%). The experimental trial's measured values showed the EE between 88.09 and 96.33%. The responses Y (R^2^: 0.9824 & PRESS: 13.71) best fit the quadratic model. This demonstrates that the proposed model can correctly forecast the 98.24% variations in responses Y. Utilizing ANOVA, the model's effectiveness was assessed (Supplementary File- Table S2).

**Table 1:** CCD formulation batches with %EE.

Batches	X_1_	X_2_	X_3_	Entrapment efficiency⁎
	*w*/w	°C	H	%
F1	+1	−1	+1	95.46 ± 1.16
F2	0	0	+1	89.05 ± 0.84
F3	0	0	−1	88.09 ± 1.30
F4	−1	0	0	92.53 ± 1.12
F5	+1	+1	+1	96.33 ± 1.20
F6	0	0	0	89.69 ± 1.32
F7	0	0	0	89.98 ± 1.06
F8	+1	+1	−1	95.4 ± 1.30
F9	0	+1	0	93.16 ± 1.24
F10	−1	−1	−1	94.52 ± 1.18
F11	0	0	0	89.94 ± 1.14
F12	+1	0	0	93.57 ± 0.84
F13	−1	−1	+1	94.33 ± 0.94
F14	+1	−1	−1	95.32 ± 1.23
F15	−1	+1	+1	95.25 ± 1.1
F16	0	−1	0	90.87 ± 1.06
F17	−1	+1	−1	94.69 ± 0.82

⁎ Values represent mean ± standard deviation (*n* = 3).

The F-value for the model, which denotes its significance, was found to be 43.39. In this instance, EE was significantly impacted by X_1_, X_2_, and the quadratic terms of X_1_, X_2_, and X_3_. For response Y, the difference between the expected R^2^ of 0.8798 and the adjusted R^2^ of 0.9597 is <0.2, indicating reasonable agreement. The polynomial Eq. (5) illustrates the relationship between independent and dependent variables as mentioned below:

(5) $$\mathrm{EE}\%=+89.58+0.4760\times {\;}_{1}+0.4330\times {\;}_{2}+0.2400\times {\;}_{3}–0.0175\;X_{1}X_{2}+0.0875\;X_{1}X_{3}+0.1925\;X_{2}X_{3}+3.68\times {{}_{1}}^{2}+2.64\times {{}_{2}}^{2}–0.8001\times {{}_{3}}^{2}$$

**Fig. 1:**
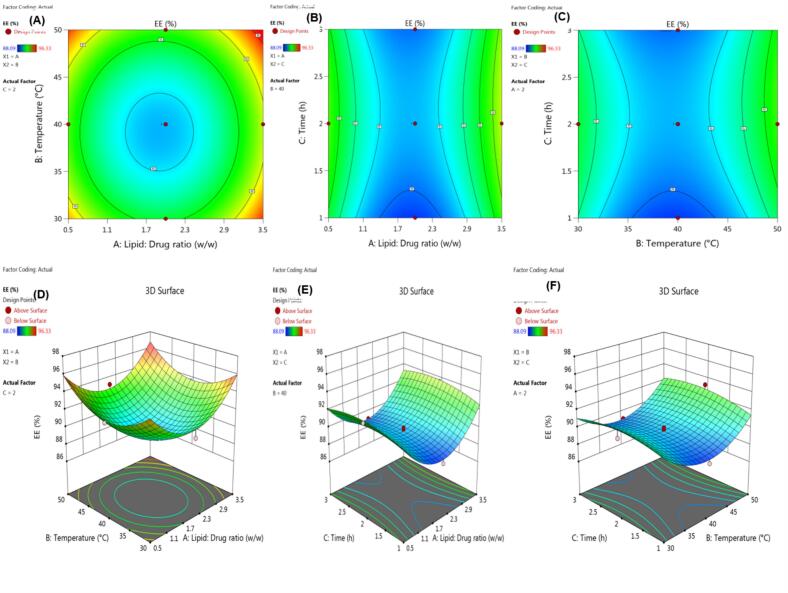
The response surface plots and the con_t_our plots of entrapment efficiency (Y, %) as a function of the ratio of Phospholipid and BSE (X_1_, w:w), the reaction temperature (X_2_, °C), and the reaction time (X_3_, h).

CCD response surfaces and contour plots are shown in Fig. 1, which displays the variation in EE for different X_1_, X_2_, and X_3_ values. Response surface and contour plots showed that the investigated factors X_1_, X_2_, and X_3_ had a significant impact on the effectiveness of EE. It was discovered that the higher EE was best suited under settings when X_1_, X_2_, and X_3_ levels were increasing. Based on these findings and the results of the multiple regression model, it was determined that the X_1_, X_2_, and X_3_ should all be set at their optimal values, which were 3.473:1, 49.72 °C, and 2.872 h, respectively.

### 3.2 Validation of the model

A further batch of BSEN was prepared to validate the developed model. X_1_, X_2_, and X_3_ values of 3.473:1, 49.72 °C, and 2.872 h generated this validation batch, which uses the model's optimal settings for the formulation and process variables. Table S3 (Supplementary File) shows the actual EE attained with the provided formulation and the anticipated efficiency of the BSEN obtained using the model. It was discovered that the typical AKBA entrapment effectiveness in naturosomes synthesized under ideal conditions was 94.87 ± 1.29%. These findings demonstrated good concordance with the model's predicted value, i.e., 96.34%, signifying the applicability and reliability of the created model. The model's relative robustness was shown by the bias (%), which was determined using Eq. (6) below and found to be <3% (1.53%) (Qin et al., 2010).

(6) $$\mathrm{Bias}\;\left(\%\right)=\mathrm{predicted value}–\mathrm{observed value}\times 100\;\mathrm{predicted value}$$

### 3.3 Drug content

Estimating the drug concentration in the finished product is a crucial component of drug-entrapped systems since it highlights the differences between formulations made under various circumstances. The drug quantity in an optimally prepared BSEN was 98.93 ± 0.78%.

### 3.4 Physico-chemical characterization of the prepared BSEN

#### 3.4.1 Photomicroscopy

**Fig. 2:**
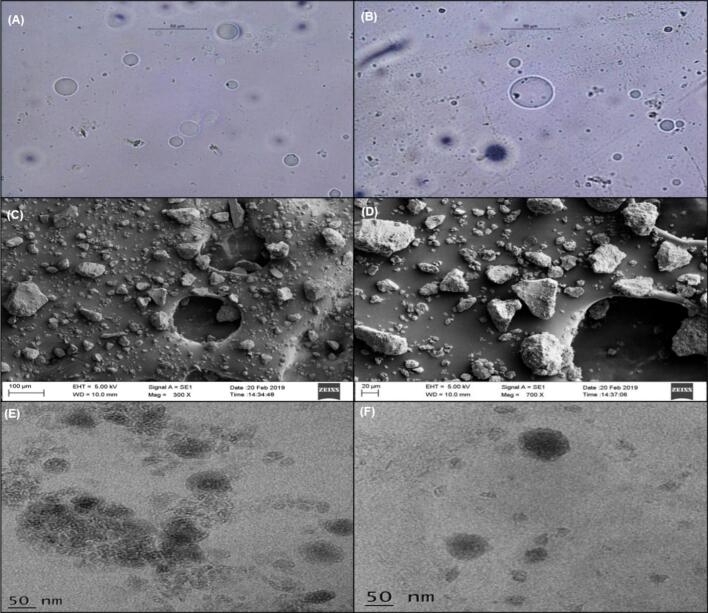
Photo microscopic images of BSEN. (A and B), SEM images of BSEN (C and D), and TEM images of BSEN (E and F).

The microscopic inspection of Figs. 2A and B revealed the presence of the complex's spherical structures. The portrayed structures looked like vesicles with the drug inside. Specifically, BSE intercalated in the lipid layers of Phospholipon®90H. The drug particles' surface shape showed that they are linked to phospholipids, which form complexes of variable sizes (Gurav et al., 2022).

#### 3.4.2 SEM

The complex was irregularly shaped and had rough surface morphology in the SEM images (Figs. 2C and D). The drug was utterly transformed into the phyto-phospholipid complex, where Phospholipon®90H physically confined it. As a result, crystals were no longer present in the complex since they had an amorphous nature (Gurav et al., 2022).

#### 3.4.3 TEM

The BSEN complex was seen on TEM images (Figs. 2E and F). A TEM investigation revealed the development of vesicular structures that looked spherical (Gurav et al., 2022).

#### 3.4.4 FTIR

**Fig. 3:**
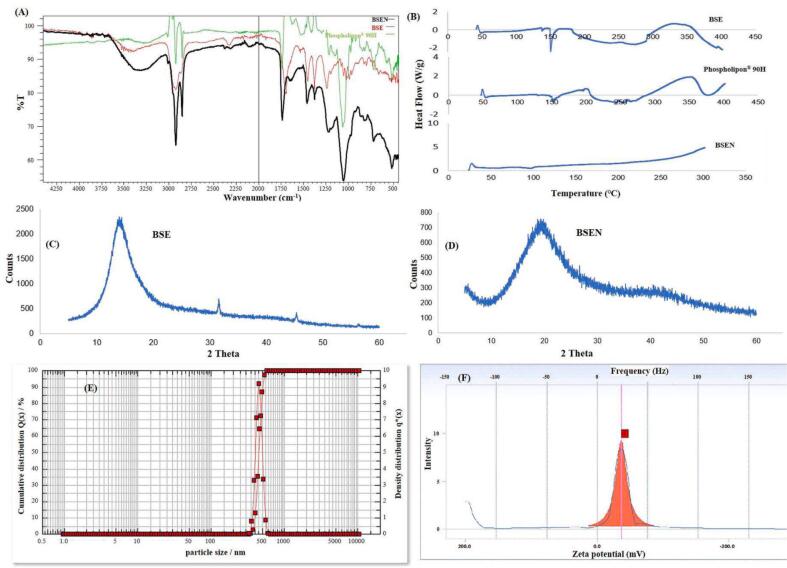
(A) FTIR spectral overlain of BSE, Phospholipon®90H, and BSEN, (B) DSC thermograms of BSE, Phospholipon®90H and BSEN, (C) X-ray diffractograms of BSE (D) (C) X-ray diffractograms of BSEN, (E) Particle size distribution of BSEN, and (F) Zeta potential analysis of BSEN.

Fig. 3A displays the findings from the FTIR examinations of the BSE, Phospholipon®90H, and the produced BSEN. The phenolic -OH stretching vibration (hydroxyl group) showed a visible peak in the FTIR spectra of BSE at 3462.22 cm^−1^. The alkyl C—H stretch in the BSE was responsible for the peak at 2922.16 cm^−1^. C=O stretching (carboxyl group) peaked at 1697.36 cm^−1^. On the other hand, the peak at 1452.40 cm^−1^ was due to the presence of C=C aromatic bending.

The typical C—H stretching signal in the long fatty acid chain was detected in Phospholipon®90H's FTIR spectra at 2918 cm^−1^ and 2850 cm^−1^, respectively. The spectrum also bears several stretching bands, including a C=O stretching band in the fatty acid ester at 1738 cm^−1^, a P=O stretching band at 1236 cm^−1^, a P-O-C stretching band at 1091 cm^−1^, and a -N+(CH_3_)_3_ stretching band at 970 cm^−1^.

Strong hydrogen bonds were formed between the -OH groups of the phospholipids and BSE in the BSEN, as evidenced by the absorption peak of hydroxyl (-OH) in the FTIR spectra of the produced BSEN, which had a broad peak with stretching moved to a lower wave number (at 3319.49 cm^−1^). Long-chain fatty acids do not play a role in naturosome formation, as evidenced by the band of the two long aliphatic chains of the fatty acids in the phospholipid molecule remains unaltered in the BSEN spectra. The P-O-C stretching vibrations altered to a lower wave number, and the P=O absorption band of phospholipids shifted to a higher wave number, respectively, confirming the development of naturosome (Gurav et al., 2022; Jena et al., 2014; Saoji et al., 2022).

#### 3.4.5 DSC

DSC is a popular tool for analyzing the interactions between various formulation components. It is common to see these interactions as the disappearance of endothermic peaks, the advent of new peaks, changes to the peak's commencement and shape, peak temperature or melting point, relative peak area, or enthalpy (Maiti et al., 2007). The DSC thermograms of pure BSE, Phospholipon®90H, and BSEN are displayed in Fig. 3B. The pure BSE showed a prominent endothermic peak at about 165.78 °C. The sharp endothermic peak for Phospholipon®90H was observed at 150.18 °C and 242.05 °C, respectively. The melting of the phospholipid most likely generated the first peak (at 150.18 °C). At 242.05 °C, a second peak is seen, possibly due to a transition from a gel to a liquid-crystalline state. Added isomeric or crystal changes to the phospholipid's carbon chain are possible (Semalty et al., 2010). Broad, partially fused endothermic peaks are visible on the thermogram of the BSEN at a temperature of 100.09 °C. The peaks of BSE and Phospholipon®90H were distinct from these. The drug's improved solubility and diminished crystallinity may be explained by decreased melting and enthalpy (Singh et al., 2013). Thus, BSEN formation was evident as the initial peaks of BSE and Phospholipon®90H disappeared from the BSEN thermogram and the lower phase transition temperature than Phospholipon®90H. These results are consistent with those found in the earlier reports of Gurav et al. (Gurav et al., 2022). DSC thermograms further suggest that the interaction between the BSE and Phospholipon®90H results from a combination of forces, including hydrogen bonds and van der Waals interactions. This interaction may indicate drug amorphization and/or complex formation (Singh et al., 2014; Zhang et al., 2010). The BSE may have interacted with the polar region of Phospholipon®90H and then been trapped in the long-chain hydrocarbon tail of the phospholipid molecule. As a result, the phase transition temperature dropped, leading to a subsequent decrease in phospholipid hydrocarbon chains and the disappearance of Phospholipon®90H's second endothermic peak (Maiti et al., 2007).

#### 3.4.6 PXRD

The PXRD patterns of BSE and BSEN are depicted in Fig. 3C and D. Sharp crystalline peaks can be seen in the BSE diffractogram. The BSEN's diffractogram showed that most of the crystalline peaks connected to the BSE had vanished. These findings corroborated earlier research in which the disappearance of drug peaks was linked to the development of drug-phospholipid complexes (Saoji et al., 2016b; Singh et al., 2014). This proved that the BSEN was formed when the BSE crystalline peaks vanished. Consequently, it can be concluded that the BSE in the Phospholipon®90H matrix may exist in either a molecularly distributed or an amorphous condition (Semalty et al., 2010).

#### 3.4.7 Particle size and zeta potential analysis

Figs. 3E and F depict the prepared BSEN's average particle size and zeta potential values. The average particle size of BSEN was 441.12 ± 32 nm. Most particles have a surface area to volume (SA/V) ratio inversely related to size. The entrapped medication is thus more readily released from the naturosome via diffusion and surface erosion when the BSEN particle is smaller and has a higher SA/V. Additionally, they benefit from allowing the drug-entrapped naturosomes to pass through physiological drug barriers. Endocytosis allows particles smaller than 500 nm to enter cells across the plasma membrane (LeFevre et al., 1978; Savic et al., 2003). However, the lymphatic system is used to uptake bigger particles (> 5 mm).

Another vital metric frequently utilized to evaluate naturosomal stability is zeta potential. The zeta potential of the prepared BSEN was −36.35 ± 1.19 mV. These findings concur with earlier research stating that zeta potential values greater than or equal to −30 mV are acceptable and indicate solid physical stability (Halarnekar et al., 2023; Sze et al., 2003).

### 3.5 Functional evaluation of BSEN

#### 3.5.1 Apparent solubility

Table S4 (Supplementary File) displays the findings from measurements of the apparent solubilities of pure BSE, PM, and a manufactured BSEN. Pure BSE was found to be poorly soluble in water (∼12 μg/ mL) but significantly more soluble in n-Octanol (∼355 μg/mL), showing that the medication has a lipophilic nature. In terms of n-octanol and aqueous solubility, PM showed no appreciable change. On the other hand, the BSEN demonstrated a substantial increase (over 16-fold) in the aqueous solubility. The drug's partial amorphization (lower molecular crystallinity) and the naturosome's overall amphiphilic character could contribute to the produced complexes' increased solubility (Singh et al., 2013; Xia et al., 2013).

#### 3.5.2 In-vitro drug release (dissolution)

**Fig. 4:**
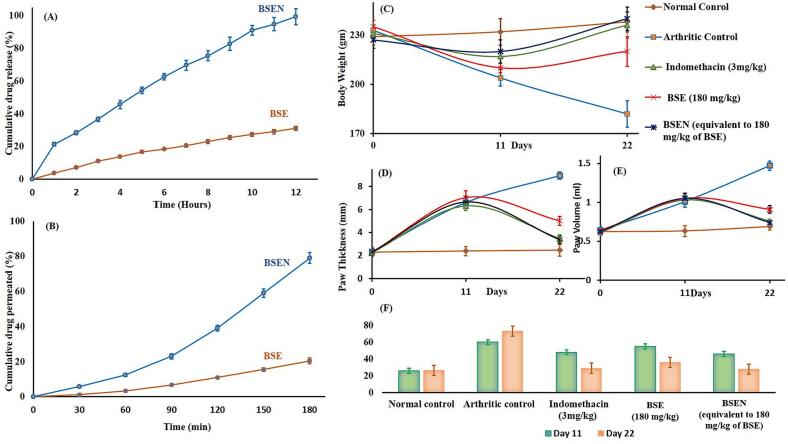
(A) In-vitro dissolution profiles of BSE and BSEN, (B) *Ex-vivo* permeability profiles of BSE and BSEN, (C) Body weight, (D) Paw Thickness, (E) Paw Volume, (F) TNF-α measurement in mice. Animals were treated with vehicle (control), Arthritic control (FCA-induced arthritis), indomethacin (3 mg/kg), pure BSE (180 mg/kg), and the BSEN (equivalent to BSE 180 mg/kg). Data expressed as mean ± SEM.

Fig. 4A displays the outcomes of *in-vitro* drug release experiments. The pure BSE demonstrated the slowest dissolution rate, with only about 31% *w*/w of BSE being dissolved at the end of the 12 h dissolution in the phosphate buffer (pH -6.8). At the end of the dissolution period, the prepared BSEN showed a noticeably faster release of BSE. Over 99% w/w BSE was seen to be released from the BSEN at the end of 12 h, indicating that the BSEN's dissolution profile followed a zero-order release. The wettability and crystal morphology of the solids have a significant impact on the dissolution rate, and the enhanced solubility and partially disrupted crystalline phase (amorphous form) in the produced naturosome may be responsible for the improved dissolution rate of BSE from the BSEN (Freag et al., 2013; Semalty et al., 2010). The increased amorphous state and enhanced water solubility of naturosomes might have contributed to the gradual accumulation of drug release.

#### 3.5.3 Ex-vivo permeability

Fig. 4B displays the outcomes of the *ex-vivo* permeability research on pure BSE and the produced BSEN using the everted intestinal method. The examined samples' permeability seems to correspond to the patterns found in the *in-vitro* release investigation. After the three-hour investigation, it was found that just 20% by weight of the pure BSE had passed through the everted intestine. However, the prepared BSEN showed a noticeably enhanced ability to pass BSE via the everted intestine. Over 79% w/w of BSE from BSEN was discovered to have permeated the biological barrier after the 3 h of testing. The amphiphilic character of the phospholipids may function as a surfactant and boost the permeability of the medication through the membrane (Li et al., 2015). This drug-phospholipid complexation technique presents itself as a viable formulation strategy for enhancing BSE delivery to the physiology due to the reported better solubility, higher dissolution rate, and observed more excellent permeability of the BSE in the produced BSEN. Our present results are coherent with Hüsch et al. (2013) study, suggesting that the poor bioavailability, absorption, and permeability of administering BSE or BAs can be enhanced by formulating into a phospholipid complex (Hüsch et al., 2013).

### 3.6 Anti-arthritic study

#### 3.6.1 Effect on body weight

Compared to normal control rats, the body weight of arthritis-affected control rats dramatically reduced (*p* < 0.01 and *p* < 0.001) from day 11 to day 22 (Fig. 4C). When compared to rats with arthritis, rats treated with indomethacin 3 mg/kg (the reference medicine) and BSEN (equivalent to 180 mg/kg BSE) showed a substantial increase in body weight (*p* < 0.05, p < 0.01 and *p* < 0.001) from days 11 to 22. However, this difference was not seen in the BSE (180 mg/kg) treatment group. The effects of BSEN and indomethacin on body weight gain were comparable (*p* > 0.05), whereas BSEN was more effective in terms of body weight gain when compared to pure BSE (p < 0.05). Wendt et al. (2019) showed that under arthritic situations, the body weights of the rats were significantly lower in the control group (arthritic rats) than in healthy or treated rats (Wendt et al., 2019). This may be linked to cachexia, a condition in which there is a decrease in food intake and a loss of muscle mass (lipolysis) that inhibits the accumulation of body mass. According to studies, cachexia in arthritic rats results from metabolic changes brought on by systemic inflammation as well as anorexia (Evans et al., 2008; Fonseca et al., 2011). Our study aligns with these reports, and treatment with BSENs ameliorated the cachexia condition, leading to improved body mass.

#### 3.6.2 Effect on paw thickness

In animals, the CFA-induced arthritis paradigm simulates chronic inflammation accompanied by multiple systemic changes, including synovial hyperplasia (Mbiantcha et al., 2017). Rats that had been given CFA (groups 2 to 5) to make them arthritic had statistically considerably (*p* < 0.01 and *p* < 0.001) thicker paws than the normal control group. When compared to BSE (180 mg/kg) in the arthritis-induced group, the indomethacin (3 mg/kg) and BSEN (equivalent to 180 mg/kg BSE) treatments dramatically (*p* < 0.05 and p < 0.01) reduced the paw thickness (Fig. 4D). Interestingly, the decreased in rat paw thickness was also reported with similar effects produced by the formulating BSEN in carrageenan-induced paw edema model (Sharma et al., 2010). This indicates that BAs developed in phytosomal delivery have dramatic anti-inflammatory benefits, which aligns with our results.

#### 3.6.3 Effect on paw volume

On day 0 before CFA injection, each rat's left hind paw volume in each group was assessed and considered as baseline values (Fig. 4E). On days 11 and 22, compared to day 0, there was a slight increase in the paw volume of the normal control. On day 11 compared to day 0, all CFA-induced arthritic groups (group 2 to 5) paw volume measurements showed a substantial increase (p < 0.01 and p < 0.001). Following medication administration, BSE (180 mg/kg) had no discernible impact on the edema, whereas BSEN (equivalent to 180 mg/kg BSE) at the same dosing level reduced the edema volume similarly as indomethacin (3 mg/kg) (p < 0.05 and p < 0.001). The decline in CFA-induced paw inflammation measures the anti-inflammatory potential of the test drug (Kshirsagar et al., 2014).

#### 3.6.4 Effect on TNF-α

After receiving a CFA injection, the rat footpad becomes inflamed around the ligaments and joint capsules. During the initial phases of inflammation, edema brought on by CFA gradually appears and persists for two weeks. Significant leukocyte infiltration, an increase in chemokine and cytokine levels, such as IL-1 and TNF-, the production of reactive oxygen species, the breakdown of cartilage and bone, and swelling and deformation are the main contributing factors (Mbiantcha et al., 2017). TNF-α is an inflammatory cytokine encoded within the major histocompatibility complex as a trimeric protein. Patients with RA have high levels of TNF-α in their blood and synovial fluid, suggesting that this may be one of the causes of RA (Farrugia and Baron, 2016). On day 11, TNF-α levels in the CFA-induced arthritic rats were higher (p < 0.01 and p < 0.001) than in the normal control group (Fig. 4F). As an inflammatory marker, TNF-α encourages inflammation and leads to the deterioration of joint tissue when its levels get elevated. When day 22 readings were compared to day 11 values, the arthritic control group's TNF-α levels increased noticeably. TNF-α levels were significantly reduced in the groups treated with indomethacin and BSEN (p < 0.05 and p < 0.001) but not in the groups treated with BSE, compared to the rats with arthritis in the control group. Literature has suggested that boswellic acid exhibits potential anti-inflammatory and anti-arthritic properties in both *in-vitro* and *in-vivo* models. The molecular target of its inhibitory activity on kappa B kinases was shown to have anti-inflammatory effects, which led to an inhibition of NF-κB activation and TNF-α release from activated monocytes (Nema et al., 2022). These findings align with our study, which suggested that boswellic acid given alone or in the form of naturosome produces potential effects in alleviating the inflammatory cascade in RA condition.

#### 3.6.5 Histopathological analysis

**Fig. 5:**
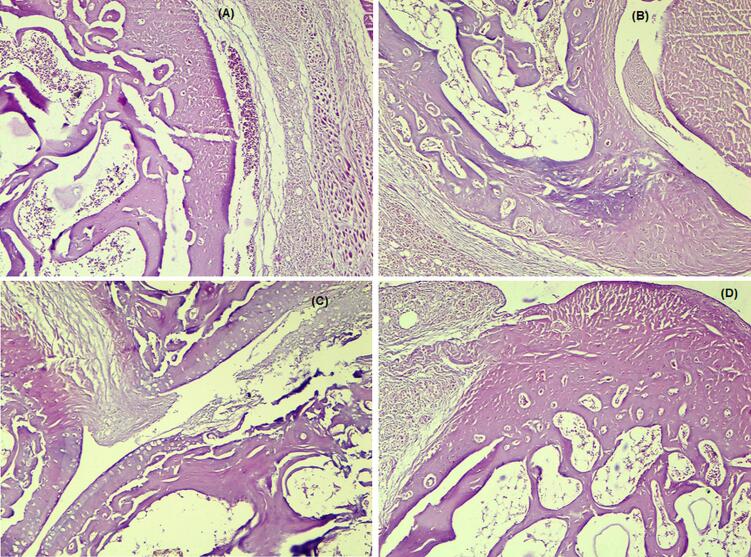
Histopathological images of the cartilage tissue of the joint sections of an inflamed hind limb. (A) Arthritic control (FCA-induced arthritis), (B) indomethacin (3 mg/kg), (C) pure BSE (180 mg/kg) and (D) the BSEN (equivalent to BSE 180 mg/kg) treated rats.

An arthritic control inflamed hind limb's joint sections' histological findings (Fig. 5A) revealed degenerative cartilage and bone tissue alterations. The cartilage tissue displayed focal areas with an uneven surface that may have been damaged or eroded. Additionally, the joint tissue showed focal infiltration of inflammatory cells. Arthritis and the degenerative characteristics of bone and cartilage were linked to pathological alterations in the cartilage tissue. Rats treated with indomethacin (3 mg/kg) (Fig. 5B) or BSEN (equivalent to 180 mg/kg BSE) (Fig. 5D) displayed normal cellular histomorphology in the bone tissue and surrounding muscle tissue in the joint sections. The bone tissue and cartilage both seemed consistent and intact. Joint tissue slices exhibited relatively fewer pathogenic or inflammatory alterations.

Rats treated with BSE (180 mg/kg) demonstrated negligible cartilage and bone tissue degeneration in the joint tissue (Fig. 5C). Only one focus with bone deterioration characteristics was visible in the cartilage tissue.

Pure BSE has limited bioavailability because of its lipophilic makeup, which may explain why it had no discernible effects on body weight, paw thickness, paw volume, or TNF-α inhibition in the current investigation. However, the same amount of BSE supplied in phospholipid nanocarrier form, i.e., BSEN, considerably raised body weight, decreased the thickness of the paw and the volume of paw edema, and inhibited TNF-α in serum, demonstrating a significantly greater bioavailability of BSE via naturosomal nano drug delivery system.

Even though both BSE and BSEN treatment showed anti-arthritic potential, BSEN appeared more effective than BSE in treating FCA-induced arthritis. The following elements may be responsible for improving BSEN's relative absorption after oral administration: BSE has a dissolving rate restriction because of its lipophilic nature, which limits its absorption and bioavailability. Interactions between the non-polar fatty acid portion of the phospholipid and the BSE may have improved BSEN's total hydrophilicity and solubility (Khan et al., 2013). As a result, the BSEN's ability to dissolve more quickly may have enhanced.

Additionally, the reduced particle size of the produced BSEN might have increased the percentage of BSE absorption following oral administration. A longer duration of effect and increased bioavailability may be brought about by the prolonged release of BSE from BSEN and a reduced metabolism (Tan et al., 2012). As noted in earlier research, intestinal transport and absorption mechanisms may have contributed to the increased BSEN oral bioavailability (Metkari et al., 2023; Saoji et al., 2016a; Yanyu et al., 2006).

### 3.7 Proposed mechanism in naturosome formation

Phospholipids are critical components of cell membranes, with a distinct structure that aids in naturosome synthesis. A phospholipid molecule comprises one hydrophilic head group and two hydrophobic fatty acid tails. In water, phospholipids spontaneously form bilayers, with hydrophilic heads facing the aqueous environment and hydrophobic tails facing inward, insulated from water. Because of their amphiphilic nature, phospholipids can form stable structures like micelles, which enclose hydrophobic compounds (Metkari et al., 2023; Saoji et al., 2017; Saoji et al., 2016b).

In the context of naturosome complex formation, the amphiphilic nature of phospholipids enables them to interact with hydrophobic bioactive compounds/extracts derived from plants. These interactions involve the hydrophobic tails of phospholipids associating with the hydrophobic regions of the bioactive compounds while the hydrophilic heads remain exposed to the surrounding medium (Gurav et al., 2022; Saoji et al., 2017; Saoji et al., 2022). This results in the formation of complexes where the bioactive compounds are encapsulated within the phospholipid bilayers. Additionally, the self-assembly properties of phospholipids contribute to the stability and uniform dispersion of the complexes (Khan et al., 2013).

In our investigation, the spectroscopic data confirmed that the interaction of phospholipid with bioactive is due to the formation of hydrogen bonds between the polar head and the polar functionalities of the active ingredient. In a nutshell, the interactions between active constituents and phospholipids occur via hydrogen bonds to generate intermolecular force rather than chemical or hybrid bonds.

## 4 Conclusion

The present investigation attempted fabrication and anti-arthritic assessment of naturosomal nanocarriers in FCA-induced arthritis. The central composite design gave the optimal conditions for preparing naturosomes by combining several rational combinations of drug: phospholipid, reaction time, and reaction temperature. As a result, naturosomal delivery of BAs enhanced their aqueous solubility (a major constraint in the therapeutic application) and dissolution rate, potentially amplifying their total therapeutic efficacy. The aforementioned findings were further confirmed by the enhanced permeability of BSEN than BAE. Also, *in-vivo* studies using the FCA-induced arthritic animal model demonstrated significantly more activity of BSEN. Further, more research examining pharmacokinetic characteristics must support the higher bioavailability and greater absorption hypotheses.

The following are the supplementary data related to this article.

Supplementary material**:** Table S1 (Supplementary File)**:** Coded levels and “Real” values for each factor under study.
Table S2 (Supplementary File)**:** ANOVA of the Quadratic model.
Table S3 (Supplementary File)**:** Comparison of the observed and predicted values in BSEN prepared under predicted optimum conditions
Table S4 (Supplementary File)**:** Solubility study of BSE, PM, and BSEN. Supplementary material

## CRediT authorship contribution statement

**Poonam Usapkar:** Writing – original draft, Validation, Software, Methodology, Investigation. **Suprit Saoji:** Writing – review & editing, Software, Resources, Investigation, Formal analysis, Data curation. **Pradnya Jagtap:** Software, Methodology, Investigation, Formal analysis, Data curation. **Muniappan Ayyanar:** Writing – review & editing, Validation, Software, Methodology, Investigation, Formal analysis, Data curation. **Mohan Kalaskar:** Writing – review & editing, Software, Resources, Methodology, Formal analysis, Data curation. **Nilambari Gurav:** Writing – review & editing, Visualization, Methodology, Formal analysis, Data curation. **Sameer Nadaf:** Writing – review & editing, Validation, Software, Methodology, Formal analysis, Data curation. **Satyendra Prasad:** Writing – review & editing, Software, Resources, Methodology, Formal analysis, Data curation. **Damiki Laloo:** Writing – review & editing, Software, Resources, Methodology, Formal analysis, Data curation. **Mohd Shahnawaz Khan:** Writing – review & editing, Validation, Resources, Funding acquisition, Formal analysis, Data curation. **Rupesh Chikhale:** Writing – review & editing, Visualization, Software, Methodology, Funding acquisition, Formal analysis, Data curation. **Shailendra Gurav:** Writing – original draft, Methodology, Investigation, Data curation, Conceptualization.

## Declaration of competing interest

The authors declare no competing interests.

## Data Availability

Data will be made available on request.
