# Supplementary material for: QbD-guided phospholipid-tagged nanonized boswellic acid naturosomal delivery for effective rheumatoid arthritis treatment
Source: Int J Pharm X. 2024 May 19;7:100257. doi: 10.1016/j.ijpx.2024.100257 (PMC11637072; doi:10.1016/j.ijpx.2024.100257)
Supplement: Supplementary file 1 — Supplementary material: Table S1 (Supplementary File): Coded levels and “Real” values for each factor under study. Table S2 (Supplementary File): ANOVA of the Quadratic model. Table S3 (Supplementary File): Comparison of the observed and predicted values in BSEN prepared under predicted optimum conditions Table S4 (Supplementary File): Solubility study of BSE, PM, and BSEN. [file mmc1.docx]

**SUPPLEMENTARY FILE**

**QBD-GUIDED PHOSPHOLIPID-TAGGED NANONIZED BOSWELLIC ACID NATUROSOMAL DELIVERY FOR EFFECTIVE RHEUMATOID ARTHRITIS TREATMENT**

Poonam Usapkar^†a^, Suprit Saoji^†b^, Pradnya Jagtap^c^, Muniappan Ayyanar^d^, Mohan Kalaskar^e^, Nilambari Gurav^f^, Sameer Nadaf^g^, Satyendra Prasad^c^, Damiki Laloo^h^, Mohd Shahnawaz Khan^i^, Rupesh Chikhale^j^, Shailendra Gurav^a*^

*^a^ Department of Pharmacognosy, Goa College of Pharmacy, Goa University, Goa -403 001, India*

*^b^ Department of Pharmaceutical Sciences, R. T. M. University, Nagpur, Maharashtra, India*

*^c^ PDEA’s S. G. R. S. College of Pharmacy, Saswad, Maharashtra, India*

*^d^ Department of Botany, A. V. V. M. Sri Pushpam College (Autonomous), Poondi (Affiliated to Bharathidasan University) – 613 503, India*

*^e^ R.C. Patel Institute of Pharmaceutical Education and Research, Shirpur, India*

*^f^ PES’s Rajaram and Tarabai Bandekar College of Pharmacy, Ponda, Goa University, Goa - India- 403401*

*^g^ Bharati Vidyapeeth College of Pharmacy, Palus 416310, Maharashtra, India*

*^h^ Department of Pharmacognosy, Girijananda Institute of Pharmaceutical Science, Girijananda Chowdhury University, Azara, Guwahati- 781017*

*^i^ Department of Biochemistry, College of Science, King Saud University, Riyadh, Saudi Arabia*

*^j^ UCL School of Pharmacy, 29−39 Brunswick Square, London WC1N 1AX, United Kingdom*

**^†^ Equal Contribution and can be treated as First Author*Corresponding Author:**

**Dr. Shailendra S. Gurav, PhD**

Department of Pharmacognosy, Goa College of Pharmacy, Panaji, Goa University,

Goa, India – 403 001, Tel- +91-9970835019,

Email: [shailendra.gurav@nic.in](mailto:shailendra.gurav@nic.in)

ORCID ID: **0000-0001-5564-2121**

**Table S1:** Coded levels and “Real” values for each factor under study.

| Variables | Levels | | |
| --- | --- | --- | --- |
|  | -1 | 0 | +1 |
| *Independent* | Real values | | |
| Phospholipid : drug ratio (X_1,_ w:w) | 0.5:1 | 2:1 | 3.5:1 |
| Reaction temperature (X_2_, °C) | 30 | 40 | 50 |
| Reaction time (X_3_, h) | 1 | 2 | 3 |
| ***Dependent*** | | | |
| Entrapment efficiency (Y, % w/w) | | | |

**Table S2:** ANOVA of the Quadratic model.

| Source | Sum of Squares | Df | Mean Square | F-value | p-value |  |
| --- | --- | --- | --- | --- | --- | --- |
| Model | 112.06 | 9 | 12.45 | 43.39 | < 0.0001 | Significant |
| X_1_-Lipid: Drug ratio | 2.27 | 1 | 2.27 | 7.90 | 0.0262 |  |
| X_2_-Temperature | 1.87 | 1 | 1.87 | 6.53 | 0.0378 |  |
| X_3_-Time | 0.5760 | 1 | 0.5760 | 2.01 | 0.1995 |  |
| X_1_X_2_ | 0.0024 | 1 | 0.0024 | 0.0085 | 0.9290 |  |
| X_1_X_3_ | 0.0613 | 1 | 0.0613 | 0.2134 | 0.6581 |  |
| X_2_X_3_ | 0.2965 | 1 | 0.2965 | 1.03 | 0.3433 |  |
| X_1_² | 36.28 | 1 | 36.28 | 126.42 | < 0.0001 |  |
| X_2_² | 18.74 | 1 | 18.74 | 65.31 | < 0.0001 |  |
| X_3_² | 1.72 | 1 | 1.72 | 5.98 | 0.0444 |  |
| Residual | 2.01 | 7 | 0.2870 |  |  |  |
| Lack of Fit | 1.96 | 5 | 0.3919 | 15.87 | 0.0603 | Not significant |
| Pure Error | 0.0494 | 2 | 0.0247 |  |  |  |
| Cor Total | 114.07 | 16 |  |  |  |  |

**Table S3:** Comparison of the observed and predicted values in BAN prepared under predicted optimum conditions

| Response Variable | Predicted Value | Observed value* | Bias (%) |
| --- | --- | --- | --- |
| Entrapment efficiency (%) | 96.34 | 94.87 ± 1.29 | 1.53 |

* Values represent mean ± standard deviation (n=3)

**Table S4:** Solubility study of BSE, PM and BAN

| Sample | Aqueous solubility (µg/mL)* | n-Octanol solubility (µg/mL)* |
| --- | --- | --- |
| BSE | 12.06 ± 0.32 | 355.13 ± 3.01 |
| PM | 13.58 ± 0.35 | 359.08 ± 3.89 |
| BAN | 191.07 ± 0.85 | 370.64 ± 4.27 |

* Values represent mean ± standard deviation (n=3)
